# 

**Published:** 1894-07-07

**Authors:** 


					Tt"l HOSPITAL ' Supplied under Royal Warrant to Her Majesty the Queen- Joly 7th. um
J T i" . m THE KING OF NATURAL TABLE WATERS. Wit 1 ? M
JoijciMiia t h e ,, tlof^anm^
n No. 406. ' Vol. XVI.
Saturday,
Julv 7th, 1894.
WITH EXTRA NURSING SUPPLEMENT. pkice twopence.
[Registered at the General Post Office as a Newspaper for Vo^treo.
transmission in the United Kingdom and Abroad.] Monthly I arts, 6d.; post free, 8d.
J  Ditto per Annum, 83.6d., post free.

				

